# Evaluation of Sirtuin 1 Levels in Peripheral Blood Mononuclear Cells of Patients With Idiopathic Pulmonary Fibrosis

**DOI:** 10.7759/cureus.30862

**Published:** 2022-10-30

**Authors:** Konstantina Deskata, Foteini Malli, Rajesh Jagirdar, George D Vavougios, Sotirios Zarogiannis, Konstantinos I Gourgoulianis, Zoe Daniil

**Affiliations:** 1 Respiratory Medicine Department, Faculty of Medicine, University Hospital of Larissa, University of Thessaly, Larissa, GRC; 2 Respiratory Disorders Lab, Faculty of Nursing and Respiratory Medicine Department, University Hospital of Larissa, University of Thessaly, Larissa, GRC; 3 Department of Physiology, Faculty of Medicine, School of Health Sciences, University Hospital of Larissa, University of Thessaly, Larissa, GRC

**Keywords:** age-related disease, aging, interstitial lung disease, tgf-β1, peripheral blood mononuclear cells, sirtuins, idiopathic pulmonary fibrosis

## Abstract

Aim: Idiopathic pulmonary fibrosis (IPF) is a devastating lung disorder that is characterized by aggressive and dysbalanced wound healing. IPF is mainly a disease of the elderly and thus is likely to share common pathophysiologic mechanisms with other more age-related diseases. Emerging evidence has linked disturbance of sirtuin-1 (SIRT1) expression and activity with aging and diseases of the elderly. In the present study, we aimed to evaluate SIRT1 expression in the peripheral blood mononuclear cells (PBMCs) of patients with IPF given the lack of studies in the literature.

Methods: We enrolled 34 IPF patients and 22 healthy volunteers (age and sex-matched). In both groups, SIRT1 levels were assessed in plasma, cell pellets of PBMCs, and supernatant from PBMCs’ culture with and without the addition of 10% human serum. We also measured transforming growth factor β1 (TGF-β1) concentration in plasma from IPF patients and controls.

Results: The mean (SD) age (years) of the healthy volunteers was 68.57±6.97 and of the IPF patients was 71.28±5.39 years (p>0.05). The mean SIRT1 concentration was found significantly decreased in the supernatant of PBMCs culture (without the addition of serum) in IPF subjects versus controls (1.97±0.59 ng/ml versus 2.40±0.74 ng/ml, respectively, p=0.047). No significant differences were observed between the two groups in the SIRT1 concentration of all the other materials. TGFβ1 concentration of IPF subjects was significantly increased when compared to controls (1281.38±2742.74 versus 131.11±156.06 ng/ml, respectively, p=0.032). Decreased SIRT1 levels in no-serum supernatant were predictive of IPF, after adjustment for age and sex (p=0.014, OR=0.124 [95%CI: 0.023-0.653]).

Conclusion: The findings of decreased concentration of SIRT1 in PBMCs supernatant and increased concentration of TGFβ1 in plasma in IPF patients versus controls provide important insights into the role of SIRT1 in IPF and could serve as a tool for the diagnosis and evaluation of patients with IPF.

## Introduction

Idiopathic pulmonary fibrosis (IPF) is a specific form of chronic, progressive, fibrosing interstitial pneumonia of unknown cause, occurring primarily in older adults and limited to the lungs, which is associated with poor prognosis [1]. Two-thirds of IPF patients are older than 60 years old at their first visit to the physician, with 66 years as the median age of diagnosis [2]. Loss of lung function is primarily attributed to overwhelming tissue remodeling, scarring, and fibrosis. The remodeling is characterized by (myo)fibroblast activation and disproportionate extracellular matrix material (ECM) accumulation [3]. Myofibroblasts facilitate wound healing, however, persistent and uncontrolled activation results in excessive ECM accumulation and contribute to the development of fibrotic disorders [4-6]. In later stages of lung injury, a variety of proinflammatory and profibrotic mediators, including transforming growth factor β1 (TGF-β1), interact to maintain fibrosis [7].

It has been proposed that IPF shares common pathophysiological mechanisms with other age-related disorders like chronic obstructive pulmonary disease and cancer [8]. Emerging evidence has linked the disturbance of silent information regulator type 1 (SIRT1) expression and activity to a variety of age-related diseases. SIRT1 is a member of a family of proteins which is predominantly a class of NAD+-dependent deacetylases, evolutionarily highly conserved [2]. Due to their indispensable role in cellular longevity through telomere maintenance and DNA repair, sirtuins are specifically referred to as anti-aging proteins. Several researchers have reported that SIRT1, SIRT3, SIRT6, and SIRT7 significantly deter the development and progression of lung fibrosis of different etiologies [2]. SIRT1 has possible implications for bleomycin-induced lung fibrosis [9].

Based on the latter observations, we hypothesize that patients with IPF may present abnormal SIRT1 levels and expression compared to controls, and we sought to assess SIRT1 levels in plasma, peripheral blood mononuclear cells (PBMCs), and PBMC culture supernatants in IPF patients and controls. Additionally, we aimed to examine the possible association of SIRT1 with a known mediator of lung fibrosis, TGFβ1.

## Materials and methods

Study population

Thirty-four IPF patients and 22 healthy volunteers participated in the study. The inclusion criteria for healthy controls were: age >60 years, a history of no smoking, or any respiratory disorder. Healthy volunteers were age and sex-matched with the patient group. IPF was diagnosed according to international guidelines [1]. The patients were recruited from the Interstitial Lung Disease Clinic of the Department of Respiratory Medicine of the University Hospital of Larisa, Greece. All participants signed an informed consent form. The study was approved by the University Hospital Ethics Committee.

Whole blood processing and PBMCs isolation

Twenty milliliters of whole blood from a peripheral vein of all the participants were collected in tubes with EDTA and processed within three to five hours for the isolation of PBMCs. Then, 10 ml of blood was then diluted with 20 ml of sterile PBS for the isolation of PBMCs as detailed below.

Twenty milliliters of Ficoll in a 50 ml Falcon tube were overlaid with the diluted blood by gently pipetting along the wall of the tube. The tube was then centrifuged at room temperature at 2500 rpms with no brakes for 20 minutes, which resulted in the formation of (1) plasma fraction, (2) buffy coat layer, (3) Ficoll-PBS interface, and (4) red blood cell pellet. The plasma fraction was collected with a 1 ml micropipette and stored at −800 °C. The buffy coat was collected with a fresh pipette tip of up to 5 ml volume and directly diluted into 20 ml of sterile PBS in a fresh 50 ml falcon tube. The diluted buffy coat fraction was subjected to a further two rounds of centrifugation at 2500 rpms with no brakes for seven minutes with the removal of the PBS supernatant. The resulting PBMC pellet was disrupted into a cell suspension with 1 ml of RPMI-1640 with no serum or antibiotic additives.

PBMCs culture

The PBMC solution was subjected to cell culture in non-adherent, sterile 12-well plates. Briefly, 0.25 ml of the PBMC suspension was added to either 1 ml of 10% human serum RPMI-1640 or to 1 ml of RPMI-1640 with no serum. The plate was incubated for 48 hours in a humified CO2 chamber. The cell suspension was then subjected to centrifugation at 5000 rpm for five minutes and further divided into pellet and culture supernatant fractions. These fractions were stored at −800 °C until further analysis for SIRT-1(ab171573) or TGF- b1(ab100647) by ELISA. The pellets were subjected to complete lysis prior to ELISA as recommended by the manufacturer's instructions.

TGFβ1 and SIRT1 measurement

Plasma and PBMC lysates were subjected to SIRT1 (ab171573) and (TGF)-b1 (ab100647) as recommended by the manufacturer’s protocol (Abcam, Cambridge, England). Briefly, capture antibody-coated wells were calibrated at room temperature prior to use. Diluted plasma (50 ml) or cell lysate (50 ml) was applied in duplicate wells, followed by the addition of detection antibody against specified antigens, and incubated in a 37 °C humidified incubator for one hour. The samples were then removed from the wells by inversion and each well was washed 3× with wash buffer (350 ml). Each well was treated and received TMB chromogenic substrate (100 ml) followed by a 10-minute incubation in the dark. The color development was then terminated by the addition of the stop solution. The plate was then read for endpoint O.D. using a spectrophotometer at 450 nm. Concentrations of SIRT1 and (TGF)-b1 were then calculated after plotting the standard curves.

Statistical analysis

Data were presented as mean ± SD unless otherwise indicated. The normal distribution was assessed by the Kolmogorov-Smirnov test. Univariate correlations were performed by Pearson’s correlation coefficient or by Spearman’s correlation coefficient according to variable distribution. Comparison between patients and controls was performed with the use of the Student’s t-test or Mann-Whitney U-test according to variable distribution. The values were statistically analyzed with an independent Student’s t-test or one-way ANOVA (for inter-group comparisons). To assess the prognostic value of SIRT1, we used multiple logistic regression analysis. GraphPad Prism software version 8.0 (GraphPad Software, San Diego, CA, USA) and SPSS 16 statistical package (SPSS Chicago, IL) were used for graphing and statistical analyses, respectively. A p-value of <0.05 was considered to be statistically significant.

## Results

The baseline characteristics of the study participants are shown in Table 1. The mean (SD) age (years) of the healthy volunteers was 68.57±6.97 and of the patient group was 71.28±5.39 years (p>0.05). Healthy volunteers and IPF patients did not differ in terms of gender (Table 1). The mean DLCO (%predicted) of the IPF patients was 40.50±15.19% (Table 1). Of the IPF group, 17.64% were current smokers and 50% were ex-smokers, while 23.52% were under long-term oxygen therapy at the inclusion of the study. Within one year following their inclusion in the study, 32.35% of the IPF patients died.

**Table 1:** Demographic and clinical characteristics of the study population. M/F: male/female, FVC: forced vital capacity, FEV1: forced expiratory volume in 1 second, DLCO: diffusing capacity of the lungs for carbon monoxide, TLC: total lung capacity, n: number of participants, IPF: idiopathic pulmonary fibrosis. *p<0.05 versus healthy volunteers.

Parameter	Healthy volunteers (n=22)	IPF patients (n=34)
Age (years)	68.57±6.97	71.28±5.39
Sex (M/F)	12/10	19/14
FVC (%predicted)	87.88±3.61	72.13±14.06*
FEV_1_/FVC (%)	84.10±2.55	82.00±6.55
DLCO (%predicted)	86.79±3.98	40.50±15.19*
TLC (%predicted)	86.22±4.90	55.38±2.71*

**Figure 1:**
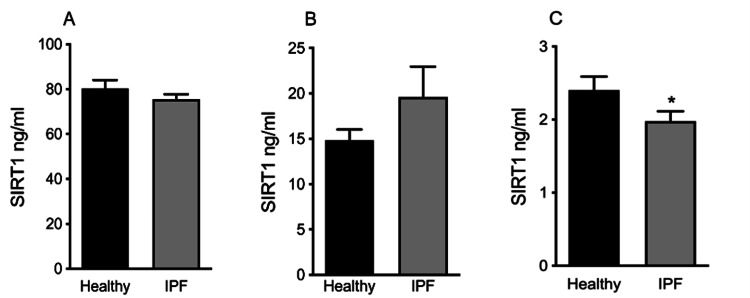
SIRT1 concentrations in A: PBMCs lysates, B: supernatant from 10% PBMCs culture, C: no serum supernatant PBMCs culture. The values are presented as mean±SE. SIRT1: Sirtuin1, PBMCs: peripheral blood mononuclear cells, IPF: idiopathic pulmonary fibrosis, ng/ml: nanograms per milliliter.

The plasma SIRT1 concentration (mean±SD) in IPF was 4.74±5.20 versus 5.48±7.76 ng/ml in healthy volunteers (p>0.05). IPF patients exhibited increased TGF-β1 concentration versus healthy volunteers (1281.38±2742.74 versus 131.11±156.06 ng/ml, respectively, p=0.032[ΚΚ1]). Next, we evaluated SIRT1 levels in PBMCs after their lysis. SIRT1 concentration in IPF was 75.5±9.28 ng/ml versus 80.34±16.49 ng/ml in controls without statistically important difference between the samples (p>0.05, Figure 1A).

We evaluated SIRT1 secretion in the supernatant that was collected after the isolation and culture of PBMCs in media with the addition of human serum 10% ("serum supernatant"), and we did not find a statistically important difference between the two groups (19.6±3.3 ng/ml in IPF versus 14.8±1.2 ng/ml in controls, p=0.34, Figure 1B). However, when PBMCs were cultured without human serum ("no serum supernatant") we observed that the SIRT1 concentration in the supernatant in IPF was significantly lower than the SIRT1 concentration of controls (1.97±0.59 ng/ml versus 2.4±0.74 ng/ml, respectively, p=0.047; Figure 1C).

We performed comparisons between all the samples (plasma, PBMCs, and PBMC culture supernatant with and without serum). In both groups, we observed a statistically important difference in SIRT1 levels between all the isolates (p<0.001). In one-by-one comparisons, all the samples differed significantly, except for the comparison between SIRT1 concentration in plasma and in "no serum" supernatant. SIRT1 concentration was higher in PBMC lysates and was decreased in descending order in the "serum" supernatant, plasma, and finally the "no serum" supernatant, respectively (Table 2).

**Table 2:** SIRT1 concentration in plasma, serum, and no serum supernatant and PBMCs’ pellets in controls and IPF patients. Data are expressed as mean±SE. *p<0.001 compared to serum supernatant, **p<0.001 compared to no-serum supernatant, ^+^p< compared to plasma, ***p<0.001 compared to PBMCs lysates. SIRT1: sirtuin1, PBMCs: peripheral blood mononuclear cells, IPF: idiopathic pulmonary fibrosis, ng/ml: nanograms per milliliter.

	Plasma	PBMCs lysate	Serum supernatant	No serum supernatant
SIRT1 (ng/ml) in controls	5.8±1.9*	80.3±3.8^+^	14.8±1.2**	2.4±0.2***
SIRT1 (ng/ml) in IPF	6.6±2.1*	75.6±2.2^+^	19.6±3.3**	2.0±0.13***

**Figure 2:**
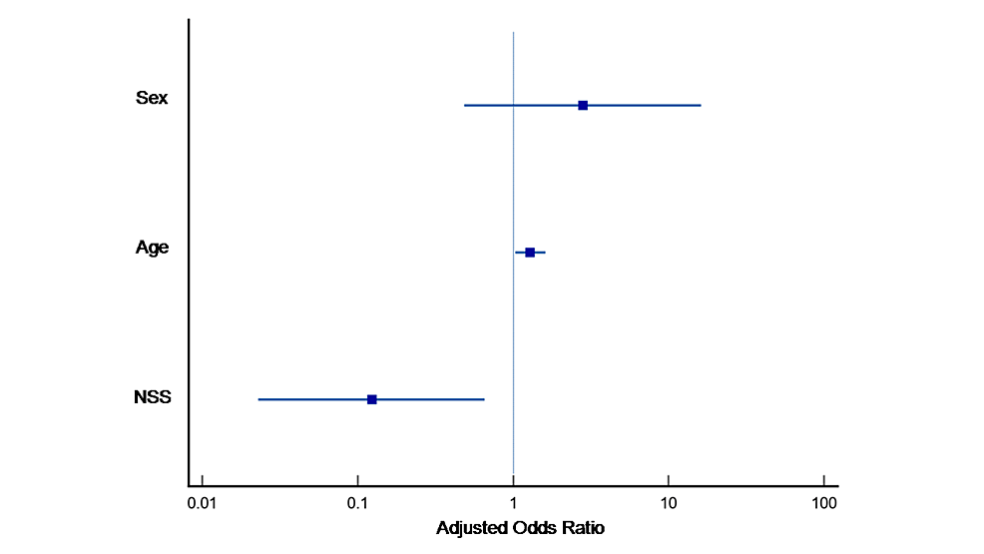
The older age and the lower SIRT1 concentration in “no serum” supernatant increase the possibility that the patient belongs to the IPF group in our sample. NSS: no-serum supernatant.

Finally, after adjusting for age and gender, increasing age was associated with an increased likelihood of exhibiting IPF (p=0.026, OR=1.289 [95%CI: 1.031-1.611]), whereas increased SIRT1 in "no serum supernatant" levels was associated with a reduction in the likelihood of IPF (p=0.014, OR=0.124 [95%CI: 0.023-0.653]) (Figure 2). This correlation was absent in the regression analysis model of the other materials (plasma, "serum supernatant," and PBMCs). This finding could indicate that "no serum supernatant" is the material of choice in measuring SIRT1 and could serve as a tool in the diagnosis and follow-up of patients with IPF.

## Discussion

In our study, we evaluated SIRT1 concentration in plasma, PBMC lysates, and PBMC culture supernatant in IPF and healthy controls. We observed statistically significantly lower mean levels of SIRT1 in the supernatant of PBMCs that were cultured in media without the addition of human serum in IPF patients compared to controls. SIRT1 was predictive of IPF when adjusted for age and sex, suggesting that SIRT1 may serve as a tool for the diagnosis of the disease. Additionally, we demonstrated increased TGF-β1 levels in IPF versus controls. To the best of our knowledge, this is the first study addressing the levels of SIRT1 in plasma and PBMCs of IPF subjects.

Sirtuins have generated considerable interest as an important player in aging biology [10]. SIRT1 is the most conserved mammalian nicotinamide adenine dinucleotide (NAD+) dependent histone deacetylase [11-12]. The association of SIRT1 with respiratory disorders has received attention in recent years. SIRT1 expression in PBMCs is downregulated in patients with bronchiectasis [13]. SIRT1 expression is decreased in healthy smokers and COPD patients compared to healthy non-smokers, while SIRT1 activity is uniquely decreased in COPD compared to both control groups [8].

During the last decade, the role of sirtuins, and especially SIRT1, in lung fibrosis has been examined. Experimental studies suggest that mice exposed to cigarette smoke demonstrate smoke-induced senescence of alveolar epithelial type 2 cells due to decreased autophagy mediated by SIRT1 inactivation. In turn, the reduction in autophagy decreased SIRT1 activity by promoting mitochondrial oxidative stress-related DNA damage [14]. Patients with systemic sclerosis-associated lung fibrosis have decreased SIRT1 mRNA in PBMCs compared to those without lung involvement [15]. There are reports in the literature that show SIRT1 is implicated in bleomycin-induced lung fibrosis. Specifically, Rozenman et al. showed that inhibition of SIRT1 activity with small molecules, such as 4-(4-chloro-2-methylphenoxy)-N-hydroxybutanamide (CMH) is a mechanism associated with cellular FLICE inhibitory protein’s (FLIP) destabilization (via Ku70 acetylation) and Fas signaling of apoptosis [16]. In these experiments, scientists also used differentiated human fibroblasts from IPF lungs and controls. Long non-coding RNAs, SIRT1 antisense (SIRT1AS AS) expression is significantly decreased in bleomycin-induced pulmonary fibrosis, whereas SIRT1AS effectively inhibits TGF-β1-mediated epithelial-mesenchymal transition (EMT) in vitro and alleviates the progression of IPF in vivo [17]. Resveratrol, an activator of SIRT1, inhibits BLM-induced lung fibrosis in mice through the reduction of EMT [9].

Despite the aforementioned reports, the literature lacks data that specifically addresses SIRT1's role in IPF. Here we reported reduced SIRT1 levels in the no-serum supernatant of PBMCs isolated from IPF subjects, while decreased SIRT1 levels were predictive of IPF, irrespective of age and sex. A plausible mechanism by which SIRT1 may be implicated in IPF is based on the role of SIRT1 in the reduction of proinflammatory cytokines. Additionally, SIRT1 may inhibit epithelial-mesenchymal transition, a key component of extracellular-matrix deposition involved in IPF pathogenesis [18]. The aforementioned observations need further confirmation in larger IPF cohorts before any definite conclusions can be drawn.

We also measured TGF-β1 in the plasma from both groups and observed increased TGF-β1 in IPF. TGF‑β promotes the fibrotic process of IPF through various signaling pathways, including Smad, MAPK, and ERK signaling, and by affecting oxidative stress [19]. The cell/tissue types which have been used in various experiments in the literature are human lung fibroblasts, mouse pulmonary fibroblasts, human fetal lung mesenchymal cells, human endothelial cells, human bronchial epithelial cells, and human alveolar epithelial cells (A549). The effect of TGF‑β1 on IPF is one of stimulation, however, there are some self‑limiting mechanisms. Wei et al. showed in their report that TGF‑β1 induces miR-133a, which inhibits myofibroblast differentiation and pulmonary fibrosis by a self-regulating mechanism. It functions as a negative feedback regulator of TGF-β1 profibrogenic pathways [20]. Our results provide further confirmation of the role of TGF-β in IPF.

As we expected, SIRT1’s concentration was higher in the PBMCs (intracellular protein) and in descending order in serum [ΚΚ1] supernatant, plasma, and no serum supernatant in both groups. Interestingly, IPF participants did not exhibit an important difference in SIRT1 concentration between PBMCs and the supernatant of PBMC culture in serum conditions. This is a finding that is suggestive of a stress condition for the PBMCs. The presence or absence of serum during cell culture plays an important role in SIRT1 production from PBMCs. As our findings demonstrate, the absence of serum during PBMCs’ culture results in a statistically important difference in SIRT1 concentration between PBMCs’ lysates and no serum supernatant but not between PBMCs’ lysates and serum supernatant. Furthermore, this difference between the healthy and IPF groups shows an increase in SIRT1 secretion from PBMCs and therefore a possible PBMCs’ disability to retain SIRT1 in the intracellular space. Another possible explanation of these results is a mutation in SIRT1 among the IPF group in a way that causes enhanced SIRT1 protein secretion during PBMC stress.

Our study has several strengths and limitations. We used a rather small cohort of IPF patients. However, one has to take into account that IPF represents a rare interstitial lung disease with an annual incidence of 10 per 100,000 people [21]. Additionally, IPF has a poor prognosis, with a mean survival of 2.5-5 years, so the pool of patients is rather small. One further limitation is the fact that patients were not longitudinally assessed for potential differences in SIRT1 and/or TGF-β1 levels.

## Conclusions

Idiopathic pulmonary fibrosis is a fatal age-related disease with an unknown etiology and without any effective medical treatment available. Most patients experience a respiratory decline with resulting respiratory failure and death within two to three years of diagnosis, and the incidence of IPF continues to rise. Consequently, it is important to investigate molecules with a possible role in IPF. We compared the concentration of SIRT1 in plasma, PBMC pellets, and supernatant of PBMCs after their culture in media with and without the addition of human serum. We found significantly reduced SIRT1 in the supernatant of PBMCs of IPF patients in comparison with the healthy group, while a lower concentration of SIRT1 in "no serum" supernatant was predictive of IPF. We also evaluated TGF-β1 concentration in plasma and we found it higher in the IPF group. Our findings support the possible implication of SIRT in IPF pathogenesis and/or diagnosis. The aforementioned observations need further confirmation with future studies.
